# Positive selection in development and growth rate regulation genes involved in species divergence of the genus *Radix*

**DOI:** 10.1186/s12862-015-0434-x

**Published:** 2015-08-19

**Authors:** Barbara Feldmeyer, Bastian Greshake, Elisabeth Funke, Ingo Ebersberger, Markus Pfenninger

**Affiliations:** Molecular Ecology Group, Senckenberg Biodiversity and Climate Research Centre (BiK-F), Georg-Voigt-Str. 14-16, Frankfurt am Main, 60325 Germany; Evolutionary Biology, Johannes Gutenberg University Mainz, Müllerweg 6, Mainz, 55099 Germany; Applied Bioinformatics Group, Institute of Cell Biology and Neuroscience, Goethe University Frankfurt, Maxvon-Laue Str. 13, Frankfurt am Main, 60438 Germany

**Keywords:** Adaptive sequence evolution, Positive selection, Transcriptomics, RNA-Seq, Mollusks, Adaptation, Reproductive isolation

## Abstract

**Background:**

Life history traits like developmental time, age and size at maturity are directly related to fitness in all organisms and play a major role in adaptive evolution and speciation processes. Comparative genomic or transcriptomic approaches to identify positively selected genes involved in species divergence can help to generate hypotheses on the driving forces behind speciation. Here we use a bottom-up approach to investigate this hypothesis by comparative analysis of orthologous transcripts of four closely related European *Radix* species.

**Results:**

Snails of the genus *Radix* occupy species specific distribution ranges with distinct climatic niches, indicating a potential for natural selection driven speciation based on ecological niche differentiation. We then inferred phylogenetic relationships among the four *Radix* species based on whole mt-genomes plus 23 nuclear loci. Three different tests to infer selection and changes in amino acid properties yielded a total of 134 genes with signatures of positive selection. The majority of these genes belonged to the functional gene ontology categories “reproduction” and “genitalia” with an overrepresentation of the functions “development” and “growth rate”.

**Conclusions:**

We show here that *Radix* species divergence may be primarily enforced by selection on life history traits such as (larval-) development and growth rate. We thus hypothesise that life history differences may confer advantages under the according climate regimes, e.g., species occupying warmer and dryer habitats might have a fitness advantage with fast developing susceptible life stages, which are more tolerant to habitat desiccation.

**Electronic supplementary material:**

The online version of this article (doi:10.1186/s12862-015-0434-x) contains supplementary material, which is available to authorized users.

## Background

Understanding the driving forces of speciation processes is a primary goal of evolutionary biology [1]. Various mechanisms can lead to the evolution of reproductive isolation between populations and ultimately to speciation [2, 3]. One of these mechanisms is ecology-driven diversifying selection imposed by the demands of adapting to niche-specific biotic and abiotic factors [3–5]. One major target of these adaptive processes is the evolution of life history traits such as lifespan, growth, development and plasticity [6, 7]. According to life history theory organisms try to optimize their survival and reproduction, thus their fitness, by following a species specific pattern in allocating resources to life history traits [6, 7]. For example developmental rates and growth are dependent on prevailing ecological conditions, and influence size and age at maturity [6, 7]. Ecological conditions affecting developmental timing and growth include predator prevalence [8, 9], nutrition [8, 10], as well as climate variables [11, 12].

Ultimately, most local environmental variables are affected by climate, which is especially the case in fresh water systems where climate is a major driver of abiotic and biotic processes [13]. Apart from the obvious impact of precipitation on freshwater systems, the temperature of a given stretch of stream or river varies seasonally, weekly and daily and is dependent on a number of factors, including air temperature, but also depth, flow, elevation, latitude, water input (overland runoff versus groundwater) and the type and extent of riparian vegetation [14, 15]. Variation in air temperature not only leads to changes in water temperature but might also lead to alterations in additional water body conditions such as reduced oxygen availability, increased salinity and the desiccation of the water body.

Due to the large number of ecological variables organisms are exposed to, it is not always possible to accurately determine the respective selective regimes acting in a system. It is thus often difficult and sometimes misleading to reconstruct *post hoc* the evolutionary forces that lead to the separation even of closely related lineages. “Reverse ecology” is one way to obtain plausible hypotheses on the driving processes of reproductive isolation [16]. This approach uses comparative genomic data to identify genes and their functions whose evolution has been driven by positive selection [16, 17]. Under the assumption that those genes most likely code for important ecological phenotypes [18] they can help to infer the response of organisms to their environment. Recent developments in next-generation sequencing technologies have led to the availability of genomic resources for many non-model organisms, allowing us to apply the reverse ecology approach to a wider range of organisms [16].

In this study we make use of the reverse ecology approach to gain insights into the role of ecological factors leading to species divergence in the pond snail genus *Radix* Montfort 1810. The genus *Radix* is part of the Lymnaeidae family (Basommatophora) and contains air-breathing, simultaneously hermaphroditic snail species, which are distributed throughout the Paleartic [19, 20]. Shell size, coloration and genital anatomy are highly variable even within species [21, 22] and often overlap between species, rendering morphological delimitation of species rather difficult [21, 23, 24]. A COI based barcoding effort based on specimen from North Western Europe resulted in five “Molecularly defined Taxonomic Units” (MOTU1-5), some of which could be attributed to existing species [23]. The type of water bodies occupied by the different species varies from deep, permanent waters (*R. auricularia*), to clear and cold streams (MOTU5), to slow flowing rivers, ditches and ponds (*R. balthica* and MOTU3) [19, 20, 23]. Moreover, the distinct distribution ranges [25] may indicate climatic niche differences among species, mirrored on the genomic level. We may thus expect to find positively selected genes with biological functions related to energy metabolism, desiccation resistance or salinity tolerance. Indeed, climate is known to affect such ecologically relevant fitness traits in other aquatic gastropods [26–28].

We use an RNA-Seq approach to obtain the transcriptomes of *R. auricularia*, MOTU3 and MOTU5 to obtain ortholog gene sequences for the selection analyses. In addition, we reconstruct the full mitochondrial genomes for each of the four species, since mitochondrial genes are known to play a central role in temperature dependent metabolism of ectotherms [29]. Three different tests for selection are applied to identify possible candidate genes involved in species divergence in the genus *Radix*.

## Results

For this study we sequenced the transcriptomes for three Central European *Radix* species. MOTU3 and *R. auricularia* were sequenced on the Roche 454 and MOTU5 on the Illumina HiSeq 2000 platform. The sequencing effort resulted in more than 400,000 to over 100Mio reads respectively. Accession numbers, summary on the number of reads, as well as summary statistics on the *de novo* assembled contigs can be found in Additional file 1. The transcriptome and assembly information of *R. balthica* has been published in a previous study [30].

### Mitochondrial genomes

**Fig. 1:**
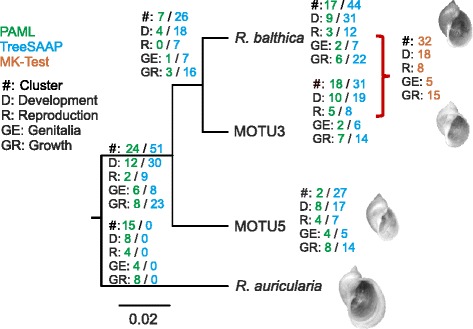
Schematic overview of the gene order in mitochondrial genomes of the four *Radix* species investigated. Gene orientation is indicated by arrows. Inferred tRNA rearrangements are indicated by arrows in the detailed section. Tree topology inferred from phylogenetic analysis (Additional file 2)

As intergenic regions in mitochondrial genomes are rather small, it was possible to assemble a large part of the mt-genomes based on transcriptome contigs. The remaining gaps were closed by additional Sanger sequences, which we also used for sequence verification. The length of the mt-genomes was 13,745 bp in *R. auricularia* [GenBank:KP098540]; 13,832 bp in MOTU5 [GenBank:KP098539]; 13,963 bp in MOTU3 [GenBank:KP098538] and 13,983 bp in *R. balthica* [GenBank:KP098541]. Mitochondrial genome nucleotide divergence ranges from 0.04 % between the two sister species *R. balthica* and MOTU3 and 0.24 % between MOTU3 and *R. auricularia*. The location and orientation of all 13 protein coding genes and the two rRNAs is identical in all four species. In contrast, the gene order within a cluster of seven tRNAs between the COII and ATP8 is variable (Fig. 1). Both observations resemble the findings of a previous comparative analysis of mt-genomes in a study of three basommatophoran genera [31]. Using *R. auricularia* as reference, the tRNA-Cys was shifted two positions towards the 5′-end of the tRNA cluster in MOTU5. Again with *R. auricularia* as reference, tRNA-Trp shifted two positions towards the 5′-end in both, *R. balthica* and MOTU3 (Fig. 1).

### Phylogenetic relationships

With *Biomphalaria glabrata* as an outgroup, both phylogenetic reconstructions lead to the same phylogenetic relationship among species (Bayesian reconstruction based on mt-genes only (Additional file 2 A); reconstruction with additional 23 nuclear loci (Additional file 2 B)). The trees consistently identify *R. balthica* and MOTU3 as sister species, as expected from Pfenninger *et al*. [23]. *R. auricularia* constitutes the earliest branching lineage among the four *Radix* species.

### Climatic niche differentiation

An analysis of 32 climatic variables [32] at 228 sampling sites of the four *Radix* species was conducted to determine climate niche differences between species. A principle component analysis revealed significant differences in PCA1 (temperature: F_(3,225)_ = 4.5; *p* = 0.005) as well as PCA2 (precipitation: F_(3,225)_ = 6.66; *p* = 0.0015) between the distribution areas of the four *Radix* species. MOTU3 inhabits the warmest and driest region, while MOTU5 can be found in the coldest habitat with the highest amount of precipitation (Additional file 3).

### Ortholog gene clusters and tests for positive selection

We used the tool *HaMStR* [http://www.sourceforge.net/projects/hamstr][ 33] to search for ortholog sequences in the *Radix* transcriptome data. For 354 genes an ortholog was detected in all four species. For an additional 236 genes we detected orthologs in each of the two sister species *R. balthica* & MOTU3.

**Fig. 2:**
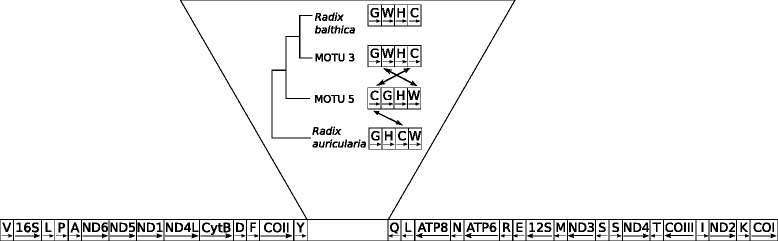
Number of selected gene clusters (#) with respective association to the four most abundant functional categories. Selected genes were identified with three different methods: *PAML* (green); *TreeSAAP* (blue); *MK-Test* (brown); D = “development” (GO:0009792/0002119/0009792); R = “reproduction” (GO:00000003/ 0018991); GE = “genitalia development” (GO:0048806/0040035); GR = “Growth” (GO:0040007/0040010); n = node# as referred to in the selection analyses. Note that a single cluster can belong to multiple functional categories. (Topology inferred from phylogenetic analysis (Additional file 2))

Three different methods (*PAML, McDonald-Kreitman Test, TreeSAAP*) were applied to test for positively selected genes among the four species. In total 134 clusters were identified as positively selected by at least one of the three methods (Additional file 4; Fig. 2). Of these, 116 were identified by a single method only, and five clusters by all three methods (Table 1). With the *PAML* method 56 clusters (out of 354 ortholog clusters) were classified as positively selected (ɷ > 1). The number of selected clusters per branch ranged from two in MOTU5 to 18 in MOTU3 (Fig. 1). There was no difference in the frequency of the four most abundant functional gene ontology categories among the branches (larval development: *X*^2^ = 1.91, df = 5, *p* = 0.86; (regulation of) growth (rate): *X*^2^ = 1.83, df = 5, *p* = 0.87; reproduction: *X*^2^ = 6.35, df = 5, *p* = 0.27; hermaphrodite genitalia development: *X*^2^ = 3.08, df = 5, *p* = 0.69), indicating their relevance in species divergence among all four species. With *TreeSAAP* 69 unique clusters fulfilled the criteria for positive selection. The number of positively selected *TreeSAAP* clusters per branch ranged from zero on the branch leading to *R. auricularia* to 44 on the branch to *R. balthica* (Fig. 1). The pair-wise comparison between the two sister species *R. balthica* and MOTU3 was based on 590 ortholog clusters, of which 32 were identified as positively selected according to the *McDonald-Kreitman Test* (*Dn/Ds* > *Pn/Ps*). None of the 134 genes with signatures of selection are of mitochondrial origin.

**Table 1:** Summary of clusters identified as positively selected by all three methods (*MK-Test* results not shown in table which automatically refer to *R. balthica* vs. MOTU3 only). n# = nodes on the phylogeny indicating the split from the common ancestor to respective species (see Fig. 2 for details)

Cluster	TreeSAAP	Paml	Annotation	GO terms
111248	n7 -- > *R. balthica*	n7 -- > Motu3	mannosyl-oligosaccharide alpha- -mannosidase isoform	determination of adult lifespan
111317	n7 -- > *R. balthica*	n7 -- > Motu3	signal recognition particle 54 kda protein	nematode larval development reproduction
	n5 -- > n6			
111407	n7 -- > *R. balthica*	n7 -- > Motu3	NA	NA
	n6 -- > Motu5	n5 -- > n6		
	n5 -- > n6			
111416	n5 -- > n6	n7 -- > *R. balthica*	dolichyl-diphosphooligosaccharide--protein glycosyltransferase 48 kda subunit	positive regulation of growth rate larval development
		n7 -- > Motu3		
		n6 -- > n7		
		n5 -- > n6		
111457	n7-- > *R. balthica*	n7 -- > Motu3	eukaryotic translation initiation factor 3 subunit e	genitalia development embryo/larval development
	n6 -- > Motu5	n5 -- > n6		
	n6 -- > n7			
	n5-- > n6			

### Functional enrichment analyses

We conducted an enrichment analysis to test for over-representation of functional categories among the complete set of positively selected genes and among subsets according to branches. The construction of clusters with *HaMStR* is based on a set of core-orthologs which are evolutionary conserved genes. The sequences/contigs used in the analyses are transcriptome based and hence a subset of all genes. We might thus introduce a systematic bias in functional categories associated to subsets of contigs. To account for these possible biases we conducted enrichment analyses with the set of contigs with and without signatures of selection against the background set of all 590 contigs each. Functional categories identified in both sets of contigs might be due to the above mentioned systematic bias and are excluded from further discussion. In the clusters without signature of selection, 14 functional categories are overrepresented (FDR < 0.05) when tested against the background set (Table 2; further details in Additional file 5). Only two categories overlap between the cluster sets with and without signatures of selection, namely “growth” and “post-embryonic development”.

**Table 2:** Summary of enriched functional categories (FDR < 0.05) overrepresented in cluster-sets with and without selection. In italic, functional categories enriched in both types of clusters

Without signature of selection	With signature of selection	
all three tests	all three tests	without MK-test
translation	*growth*	regulation of growth
cellular protein metabolic process	regulation of growth	*growth*
*growth*	positive regulation of growth	positive regulation of biological process
cellular process involved in reproduction	positive regulation of biological process	positive regulation of growth
mitochondrial ATP synthesis coupled electron transport	positive regulation of growth rate	positive regulation of growth rate
*post-embryonic development*	regulation of growth rate	regulation of growth rate
meiotic cell cycle	nematode larval development	
ribosome	larval development	
ribonucleoprotein complex	*post-embryonic development*	
cytoplasmic part		
cytoplasm		
macromolecular complex		
structural constituent of ribosome		
structural molecule activity		

Excluding the two overlapping functional categories, seven categories are exclusively overrepresented in the complete selected cluster set (all four species; all three selection tests), which are mainly involved in the regulation of growth (-rate) and larval development (Table 2). When excluding the MK-test identified genes based on MOTU3 and *R. balthica* only, the development related functions dropped out, but the growth (-rate) related functional categories are still overrepresented in this smaller cluster-set (all four species; PAML + TreeSaap) (Table 2). In the cluster set with the 32 selected genes identified between *R. balthica* and MOTU3 by the MK-Test, none of the functions appears to be overrepresented.

With respect to our initial hypothesis that genes involved in metabolism could play a role in species divergence, we could not identify any metabolism related overrepresented functional category in the selected gene clusters. However, out of the 134 genes with signs of positive selection, seven are involved in metabolism related functions such as “oxidation reduction process” and “metabolic process”. Three of these seven show signs of selection on the branch to *R. balthica*, and two more between *R. balthica* and MOTU3 by the MK-Test. Thus only two of the seven genes are found on branches other than *R. balthica*, namely *R. auricularia* and the branch leading from *R. auricularia* to the other species.

## Discussion

Understanding the underlying forces leading to species divergence is of great interest in evolutionary biology. One possible mechanism is ecological speciation, defined as the evolution of reproductive isolation between populations as a result of ecologically-based divergent natural selection [5]. We set out to determine genes possibly involved in species divergence among four freshwater snail species of the genus *Radix* inhabiting distinct distribution ranges. We hypothesized that the distribution ranges differ in climatic niche properties, leading to species divergences based on selection on species specific physiological traits. We show here, that indeed distribution ranges of the four species differ significantly in their climatic conditions, mainly in precipitation and temperature. However, genes relevant in physiology and metabolic related processes only seem to play a minor role.

As mitochondrial genes are major players in the respiratory cascade and are thus involved in metabolic processes [34], selection on some of these genes involved in oxidative phosphorylation may lead to adaptation to different environments [29, 35, 36]. The comparison of the four mitochondrial genomes revealed rather large congruence among species. While the gene order of protein coding genes is identical among species, the position of four tRNAs (GWHC), within a cluster of seven tRNAs varies between species. Even though the *Radix* species investigated here inhabit distinct climatic niches, none of the mitochondrial genes, central in energy metabolism, shows signatures of selection among the *Radix* species.

### Function and relevance of nuclear genes with signs of positive selection

In contrast to the mitochondrial genes, we were able to identify 134 nuclear genes with signs of positive selection between the four species. Seven of these genes are related to metabolism and oxidation processes and thus might indicate physiological adaptations in respect to temperature differences. Many of these were detected between the two sister species *R. balthica* and MOTU3. This corroborates findings of a recent study investigating a hybrid zone between these two species, where precipitation and temperature were the environmental factors explaining species and hybrid zone distribution [37]. Amongst the genes with signs of positive selection we also identified functions related to reproduction and hermaphrodite genitalia development. Genitalia morphology is one of the most species specific characteristics, and plays a major role in species divergence [38–40]. Genitalia morphology is known to be subjected to rapid evolution through sexual selection [41], which is especially wide spread in animals with internal fertilization [39]. Our findings might indicate a role of ongoing active enforcement of mechanical reproductive isolation in the *Radix* complex, albeit considerable inter- and intra-specific morphological variations for the bursa position and bursa duct [21, 42].

However, the most frequent and overrepresented functions among the selected genes are larval development and (regulation of-) growth (-rate). These functions are well known life-history traits. Based on life-history theory, it is generally assumed that organisms are facing numerous trade-offs due to a limited amount of resources [6, 7]. For example, long lived organisms usually produce only a small number of offspring, whereas short lived organisms produce comparatively many. In general, body size is positively correlated with survival and fecundity, however larger size often requires additional time for growth, thus leading to older age at maturity [6, 7]. On the other hand, when the risk of mortality increases in the larval environment, faster development at the cost of smaller body size may still be favored [6, 43]. Thus depending on the available resources and environmental conditions, organisms need to trade off which of the trajectories they will follow.

With respect to larval development, two factors play a crucial role; timing and rate. Developmental timing (also called heterochrony) is a complex trait [44]. It can lead to shifts in timing of gene expression and result in morphological alterations [44, 45], even though the underlying genes are shared among distantly related groups [46]. In fact, recent publications on several gastropod species, and *Radix* in particular, have uncovered inter- and intraspecific variation in embryonic development [47, 48], and a correlation between developmental timing and genetic differentiation in *R. balthica* populations [48]. Thus the variation in developmental timing in combination with genetic differentiation could serve as raw material for natural selection and drive species divergence [48].

Developmental rate, the time elapsed from embryo to the reproductive phase, shows considerable variation in natural populations [49]. Developmental rate genes have pleiotropic effects on several adult traits and the action of most of them is sensitive to temperature during development [50]. In this respect the role of environmental conditions has been suggested as a selective force driving the differentiation of embryonic development in a number of species, such as nematodes [51, 52], lizards [53] and various gastropod taxa [54]. Interestingly, developmental rates are under strong selection in a number of aquatic organisms facing the threat of desiccation, since it allows organisms and their live stages to avoid unfavorable conditions [55, 56]. For example Killifish embryos decrease their developmental rates leading to a developmental arrest in early stages of development in response to desiccation [56]. On the other hand, many amphibians accelerate development to avoid desiccation, often at the cost of body size [55 and authors therein]. In gastropod species life stage, as well as individual size have a large intra-specific effect on desiccation resistance [57–59]. In snails, an increase in shell size can be a mechanism of desiccation resistance due to reduced water loss as a result of a better surface-volume ratio [58, 59]. From these examples it becomes clear that the interplay between developmental timing, the rate of development, growth rate and size may indeed play an important role in shaping the fitness landscape of aquatic organisms.

As mentioned before, the hybrid zone between the sister species *R. balthica* and MOTU3 is best explained by local climatic factors, namely precipitation and temperature [37]. The great number of positively selected genes with functions in development and growth (-rate) together with the above examples and the pronounced climatic differences between the distribution areas of the four *Radix* species, leads to the hypothesis that species divergence is mainly driven by a trade-off in life-history parameters leading to niche adaptation. For example in hot and dry environments a quick development with fast growth might prevent susceptible larvae and small individuals from desiccation. Cold environments, on the other hand, might lead to slow development, smaller shell and smaller clutch sizes, however might result in longer lifespan [60].

## Conclusions

Based on pronounced differences in climatic niche properties between the four *Radix* species, we expected to find genes involved in physiological and metabolic traits to be under selection. However, the results from our study suggest that species divergence in *Radix* is mainly driven by selection on life-history traits, mainly development and growth, rather than physiological properties. As climate is known to have direct effects on various water body properties and therefore also on ecologically relevant fitness traits in aquatic gastropods, such as desiccation tolerance [26–28], we hypothesize that developmental rate and individual size might be of high adaptive importance in the genus *Radix*. The bottom up approach applied here provides a number of unexpected results and new hypotheses, which can now be tested in follow up functional experiments. This reverse ecology approach has thus proven valuable for hypotheses generation in evolutionary research.

## Methods

### Sample collection and treatments

Snails for transcriptome analyses were originally collected from different sites in France (MOTU3), Switzerland (MOTU5) and Germany (*R. auricularia*) between 2005 and 2011 (Additional file 6) and kept in water basins untill the start of this project. Information on treatment and sequence generation for *R. balthica* snails is reported in a previous study and documented in Feldmeyer et al. [30]. We placed individual snails in different temperature treatments. In 2011 snails were transferred to 500 ml glass jars and underwent one of six treatments, with two individuals per population, per treatment: Three days at 10 °C or 30 °C with and without aeration, 10 min temperature shock at either 4 °C or 36 °C before storage in RNA*later* (Qiagen). RNA was isolated following the RNeasy maxi kit protocol (Qiagen). cDNA production, normalization, and sequencing was performed by GenXPro GmbH (Frankfurt, Germany). Due to technical issues and different time points of sequencing, the three libraries were sequenced on different platforms, MOTU3 + *R. auricularia* on the 454 FLX system, and MOTU5 on an Illumina HiSeq 2000. Since we were interested in sequence divergence between species, the usage of different platforms will not impair data analyses as might be expected for gene expression studies.

### RNA-sequencing and read processing

For the 454 reads of MOTU3 and *R. auricularia* the script sff_extract (https://bioinf.comav.upv.es/sff_extract/) version 0.3.0, part of the MIRA assembler [61] was used to trim fragments according to the parameters determined by the 454 sequencing pipeline of Roche. MIRA, version 3.4.0, was used for the assembly of the 454 data sets, as simulation results with in silico generated 454 reads show it is one of the best assemblers for the *de novo* assembly of transcriptomes [62]. MIRA was run using the –accurate quick flag, additionally using the –AS:sep flag to force skimming and recalculating of the Smith-Waterman alignments after each main pass of the algorithm. This was used in combination with the quick settings for the *de novo* assembly of 454 transcriptomes, –denovo, 454, est. For the manually trimmed instances, where no trace data was available, the option –notrace was used as well.

Adaptor removal and *de novo* assembly for the Illumina generated reads of *R. balthica* and MOTU5 were conducted using the software CLC Genomics Workbench v3.7 (CLC Bio). The CLC Genomics Workbench uses a de Bruijn graph-based method that is suitable for the efficient assembly of short read data sets with large numbers of reads. For the assembly the bubble size was set to 50 and the kmer size was chosen automatically resulting in a size of 22 for *R. balthica* and 24 for MOTU5.

### Mt-genome assembly, verification and annotation

To reconstruct the mitochondrial genomes for MOTUs 3, 5 and *R. auricularia,* each of the RNAseq read sets were searched against the *R. balthica* mt-genome [31] using BlastN. Reads with mt-genome hits were assembled in GENEIOUS v4.8.5 (Biomatters). To close gaps, as well to confirm the accuracy of the assembly, primers were designed using the PRIMER3 web interface (http://primer3.sourceforge.net/webif.php) (Additional file 7). Due to possible sequencing and assembly errors, we re-sequenced the mt-genomes of all four species, including *R. balthica,* with the Sanger method. The annotated *R. balthica* mt-genome [31] and pulmonate gene- and rRNA-sequences published in White *et al*. [63] were used as reference for gene prediction. tRNAs were predicted using the softwares tRNA-scanner [64] and ARWEN (Laslett&Canbäck 2008). Where necessary, missing tRNAs were annotated manually by alignment of pulmonate tRNA-sequences [63].

### Construction of ortholog clusters

The ortholog search was conducted with *HaMStR* v9 [34; http://sourceforge.net/projects/hamstr]. *HaMStR* comes with a set of 1253 so called core-orthologs, i.e., a set of pre-computed clusters of ortholog genes of species with a complete genome available and known phylogeny (species included: *Lottia gigantean, Helobdella robusta, Capitella capitata, Schistosoma masoni, Apis mellifera, Daphnia pulex* and *Caenorhabditis elegans*). While the number of possible ortholog-clusters is limited using this program, the main advantage is the small number of false positives [33]. Open reading frames were predicted using *genewise* [65], a Hidden Markov Model based program which accounts for frameshifts wrongly introduced by sequencing errors using standard parameters with flags set to –*trans* –*cdna* –*pep* –*sum*. The predicted protein sequences inside each ortholog cluster were aligned using MUSCLE v3.8.31 [66] with standard parameters. These protein alignments were then used as a reference for creating codonwise alignments of the protein encoding nucleotide sequences using a custom Python script, utilizing *BioPython* [67]. The UTRs of each ortholog cluster were directly aligned using MUSCLE. For all subsequent analyses only sequence alignments with a mean nucleotide sequence divergence of < 10 % between all four species were analyzed to reduce false positives.

### Phylogenetic analysis

Evolutionary relationships among the four *Radix* species were reconstructed based on the complete mitochondrial genomes and 23 nuclear loci. The latter were chosen based on annotated gene clusters, which added up to a length of 14.836 bp, comparable to the mt-genomes (13.745 bp - 13.963 bp). To include *Biomphalaria glabrata* as outgroup, the 23 nuclear loci were searched against the *B. glabrata* gene set provided by VectorBase (https://www.vectorbase.org/) using BLASTx. The *B. glabrata* mt-genome was obtained from Genbank (accession: AAQ75773.1). The sequences were aligned to the ortholog *Radix* clusters using BioEdit [68]. MEGA5.4 [69] was used to identify the best fitting model of nucleotide sequence evolution for the mt-genomes and each of the 23 nuclear loci (Additional file 8). We built a Bayesian tree based on the mt-genomes only and one based the mt-genomes plus the nuclear loci using Beast v1.8.0 [70]. This program uses multiple loci to infer a species tree that takes into account stochastic differences in the coalescent histories of the sampled gene genealogies. The analysis was performed with a Yule prior, and run for 10.000.000 generations, sampling every 1000 generations for a total of 10.000 trees. To assess convergence, the effective sample size values and consistency of parameter estimates were monitored using Tracer v1.5 (http://tree.bio.ed.ac.uk/software/tracer/). From the 10.000 sampled genealogies, the maximum-clade credibility tree was obtained using TreeAnnotator in BEAST, discarding the first 1.000 trees as burn-in.

### Selection and functional divergence analyses

Tests for positive selection were performed using three different methods and tools: a) *PAML* v4.7 [71], b) a custom version of the *McDonald-Kreitman Test* implemented in Python using features of the *BioPython* package (http://www.biopython.org) and c) the program *TreeSAAP* v3.2 [72]. *PAML* was used to detect positive selection amongst all four species, while the McDonald-Kreitman test was performed on an additional 236 ortholog clusters containing sequences from *R. balthica* and MOTU3 only. In addition, we used *TreeSAAP* to determine the magnitude of changes in the physiochemical properties of the amino acid resulting from non-synonymous substitutions among the four species.

To detect signatures of positive selection the program *codeml* of the *PAML* package was used to perform a branch model test. *codeml* estimates the nonsynonymous/synonymous substitution ratio (ɷ = dN/dS), where ɷ = 1 indicates neutral evolution, ɷ < 1 purifying selection, and ɷ > 1 indicates positive selection. Calculation of global ω values using the one ratio model was followed by calculation of branch-specific ω using the free ratio model. Each model assigns a log-likelihood to each examined alignment and tree topology. To test for statistical significance log-likelihood ratios were calculated and FDR corrected for multiple testing [73]. The tree topology as inferred by the phylogenetic analyses was used as input for *codeml* (Additional file 2). This method is quite robust for between species comparisons as it is based on the rate of nonsynonymous to synonymous substitutions [74–76]. Excess nonsynonymous substitutions, accumulate only slowly by sequential selective sweeps [74]. Thus between population variation within species will only have minor effect on the identification of differentially selected genes among species.

Instead of comparing the rates of synonymous to non-synonymous substitutions, the *McDonald-Kreitman Test* (MK test) looks into the absolute number of fixed, non-variable, substitutions *D* and variable polymorphisms *P* and into whether those substitutions and polymorphisms are synonymous (*Ds* and *Ps*) or non-synonymous (*Dn* and *Pn*). The principle idea underlying this test is the following: Given neutral evolution, the ratio of non-synonymous to synonymous polymorphisms *Pn/Ps* is expected to be the same as the ratio of non-synonymous to synonymous substitutions *Dn/Ds* [77]. To obtain substitution and polymorphism information, a custom python script was written to read a pairwise alignment in FASTA format and iterate over each codon present in the alignment. SNPs were called when the read depth at the position of interest was at least 10 and the minor allele frequency was 0.2 without a cutoff for the required base qualities of the reads. Codons with gaps or missing data were ignored. If a given codon differed between both species, it was classified as either non-synonymous (*n*) or synonymous (*s*) and either as a polymorphic site (*P*) or a substitution (*D*). From these raw counts, the *Dn/Ds* and the *Pn/Ps* ratios were calculated. Positive selection was assumed if *Dn/Ds* > *Pn/Ps*.

*TreeSAAP* tests the effect of amino acid changes for up to 31 different physiochemical properties, like bulkiness or polarity. The observed changes and their impact are compared to a distribution expected at random under neutral conditions, with significance determined through a goodness-of-fit test under a *χ*^2^ distribution [72]. *TreeSAAP* was run with the ortholog clusters concatenated in chunks of 100 clusters per run. In each run, all 31 properties were tested with a window size of 20 codons and the same tree as used for *PAML*. Again, only clusters with a mean sequence divergence < 10 % were used. To avoid false positives due to sequencing or assembly errors, substitutions were classified as significant when at least two codons were predicted to have an impact class of ≥ 6 for the physiochemical property changes with a p-value of < 0,001.

### Annotation and enrichment analyses

All core ortholog-clusters provided by *HaMStR* are provided with unique gene identifiers based on the *C. elegans* WB190 genome. The according annotation file was downloaded from *the Gene Ontology* website (http://www.geneontology.org/). We used a custom Python script implemented in the *goatools* package (https://github.com/tanghaibao/goatools) to extract all GO terms and the corresponding descriptions from the sub-ontology “biological process” and to map this information to the ortholog clusters. The 1252 *HaMStR* core ortholog sequences were used as “neutral” reference data set for the enrichment analyses, and various cluster sets as test-set (with signature of selection: all 134 clusters, 112 clusters based on *PAML* and *TreeSAAP* including all four species, and 23 *MK-Test* clusters based on *R. balthica* and MOTU3 only; without signature of selection: 456 *R. balthica* and MOTU3 only; 235 clusters containing sequences of all four species). A one-tailed Fisher’s Exact Test was performed in *BLAST2GO* [78], with a p-value cut-off of 0,05, after FDR correction for multiple testing [73]. If many of our selected genes are indeed involved in specific functions, we would expect to find these overrepresented in the set of selected genes, but not so in the set of genes without signatures of selection, independent of possible biases. To exclude the possibility of a methodological bias, we not only tested for enriched functional categories in clusters with signatures of selection but also without selective signatures. A possible bias could have resulted from the reference data set (*HaMStR* core orthologs are evolutionary conserved genes) or the nature of transcriptome obtained contigs, which “only” represent a subset of genes expressed at the time of RNA isolation.

### Inference on climatic niche

To assess climatic niche divergence between the four *Radix* species, we extracted 35 climatic parameters (e.g., precipitation, various temperature and Bioclim parameters) for each of the 225 sampling points for the period from 1960 - 2000 from publicly available WorldClim data, incorporated in DIVA-GIS [79]. We used a principle component analysis (PCA) based on a correlation matrix to contrast the most important climatic factors between the species niches in Past 3.01 [80].

### Data accessibility

All raw sequence data are deposited as BioProject [GenBank:PRJNA265216]. Ortholog clusters can be retrieved from Dryad [doi:10.5061/dryad.1d49q]. All other data are deposited as supporting information.

## Additional files

Additional file 1:
**Results of the a) sequencing effort and b) assembly statistics.** (PDF 257 kb) Additional file 2:
**Phylogenetic inference on species relationships.** (PDF 255 kb) Additional file 3:
**Results and figure for the climate niche divergence PCA.** (PDF 14 kb) Additional file 4:
**Selected genes with annotations.** (XLSX 35 kb) Additional file 5:
**Results enrichment analyses.** (XLSX 11 kb) Additional file 6:
**Snail collection sites.** (PDF 330 kb) Additional file 7:
***Radix*** **mt-genome** **primers.** (XLS 36 kb) Additional file 8:
**Annotations and evolutionary models of the clusters used for phylogenetic inference.** (XLSX 10 kb)
