# Supplementary material for: Positive selection in development and growth rate regulation genes involved in species divergence of the genus Radix
Source: BMC Evol Biol. 2015 Aug 19;15:164. doi: 10.1186/s12862-015-0434-x (PMC4539673; doi:10.1186/s12862-015-0434-x)
Supplement: Additional file 1: — Results of the a) sequencing effort and b) assembly statistics. (PDF 257 kb) [file 12862_2015_434_MOESM1_ESM.pdf]

Additional file 1

Results of the a) sequencing effort and b) assembly statistics

a) Results of the sequencing runs for the three *Radix* species, newly sequenced in this study.

Species	Technology	Number of reads	Mean read length	Short read archive accession
MOTU 3	454 FLX	705.198	380.13	SRX965814
R. auricularia	454 FLX	427.636	345.41	SRX965774
MOTU 5	Illumina HiSeq 2000	107.406.129	72.55	SRX965818

b) Summary of the *de novo* assemblies for the four *Radix* species (*R. balthica* reads were obtained from a previous study (Feldmeyer *et al*. 2011)).

species	# of contigs	# of reads	n50	size min	size max
R. balthica	52.186	9.197.902	326	58	2.290
MOTU3	49.334	538.796	804	40	10.042
R. auricularia	27.360	325.953	771	40	10.076
MOTU5	278.901	92.356.656	351	59	36.585
