# Supplementary material for: Positive selection in development and growth rate regulation genes involved in species divergence of the genus Radix
Source: BMC Evol Biol. 2015 Aug 19;15:164. doi: 10.1186/s12862-015-0434-x (PMC4539673; doi:10.1186/s12862-015-0434-x)
Supplement: Additional file 2: — Phylogenetic inference on species relationships. (PDF 255 kb) [file 12862_2015_434_MOESM2_ESM.pdf]

Additional File 2

Radix Phylogeny

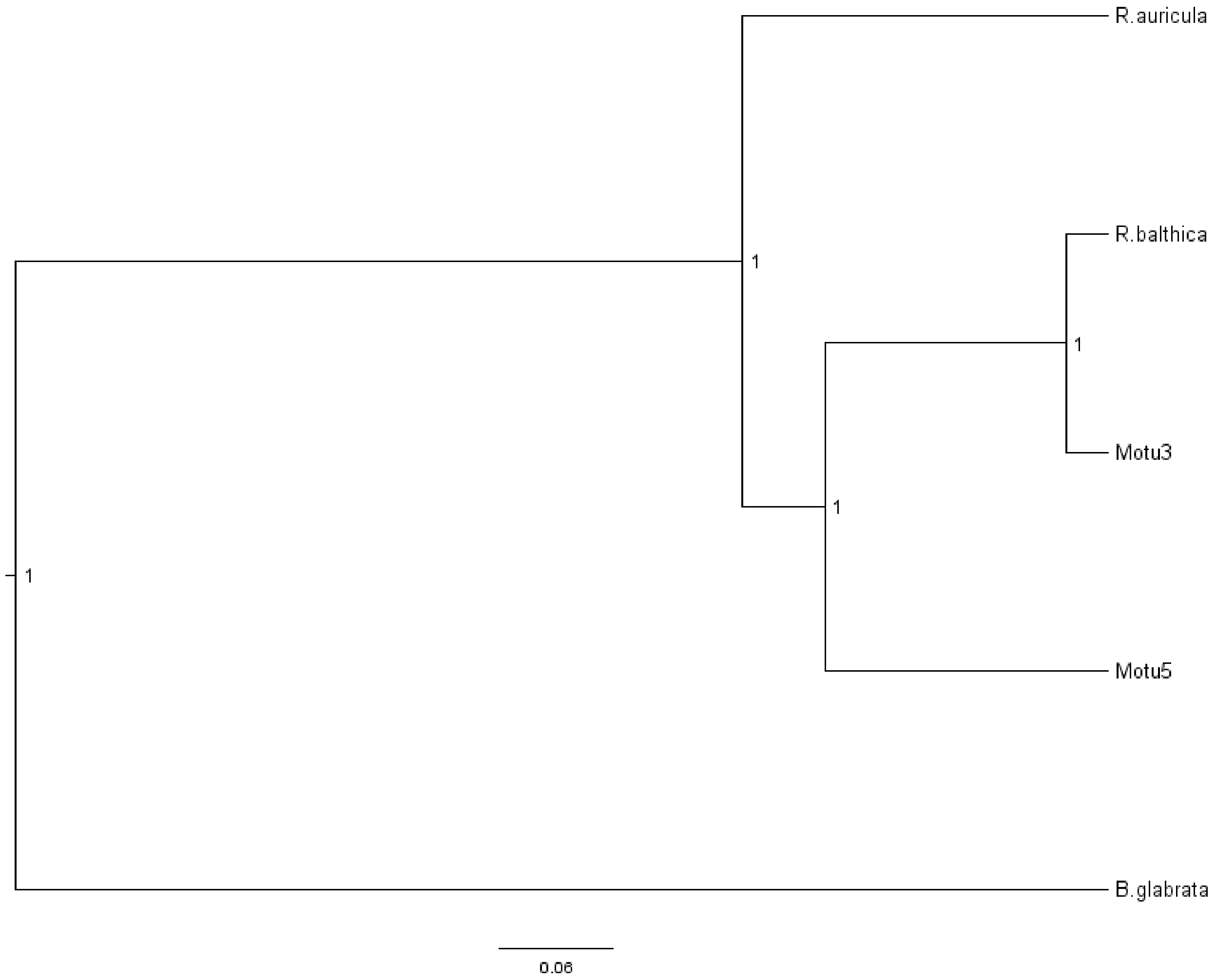

Figure S2a: Bayesian phylogeny of the four Radix species with *Biomphalaria glabrata* as outgroup based on the mt-genome only. Posterior probabilities indicated at the nodes.

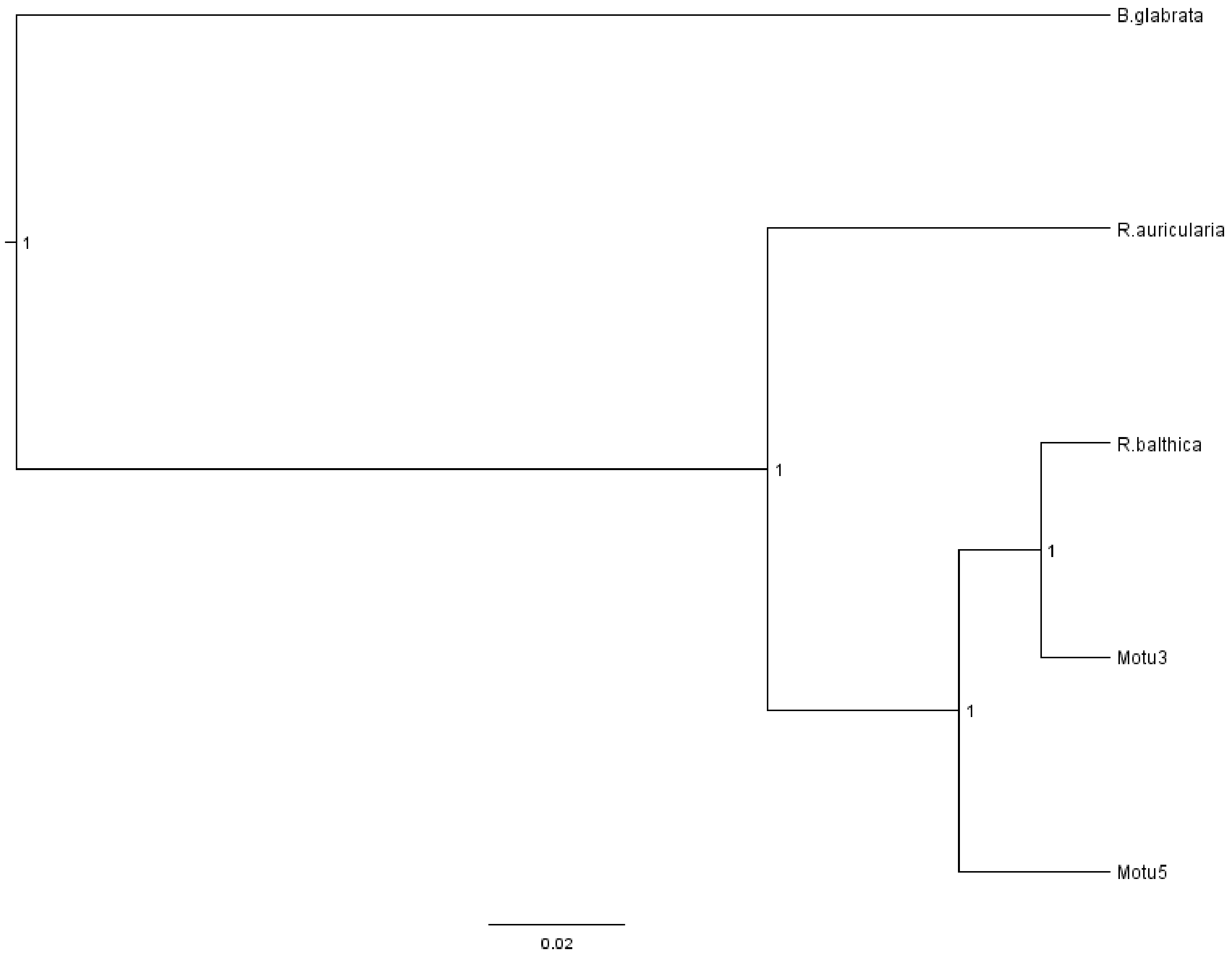

Figure S2b: Bayesian phylogeny of the four Radix species with *Biomphalaria glabrata* as outgroup based on the mt-genome as well as 23 additional loci. Posterior probabilities indicated at the nodes.
