# Supplementary material for: Positive selection in development and growth rate regulation genes involved in species divergence of the genus Radix
Source: BMC Evol Biol. 2015 Aug 19;15:164. doi: 10.1186/s12862-015-0434-x (PMC4539673; doi:10.1186/s12862-015-0434-x)
Supplement: Additional file 3: — Results and figure for the climate niche divergence PCA. (PDF 14 kb) [file 12862_2015_434_MOESM3_ESM.pdf]

Additional file 3

Climate PCA

A PCA based on 31 climatic variables indicates strong climatic niche divergence between the four snail species investigated in this study on PCA1 (temperature: $F_{(3,225)} = 4.5$, $p = 0.005$) as well as PCA2 (precipitation: $F_{(3,225)} = 6.66$; $p = 0.0015$).

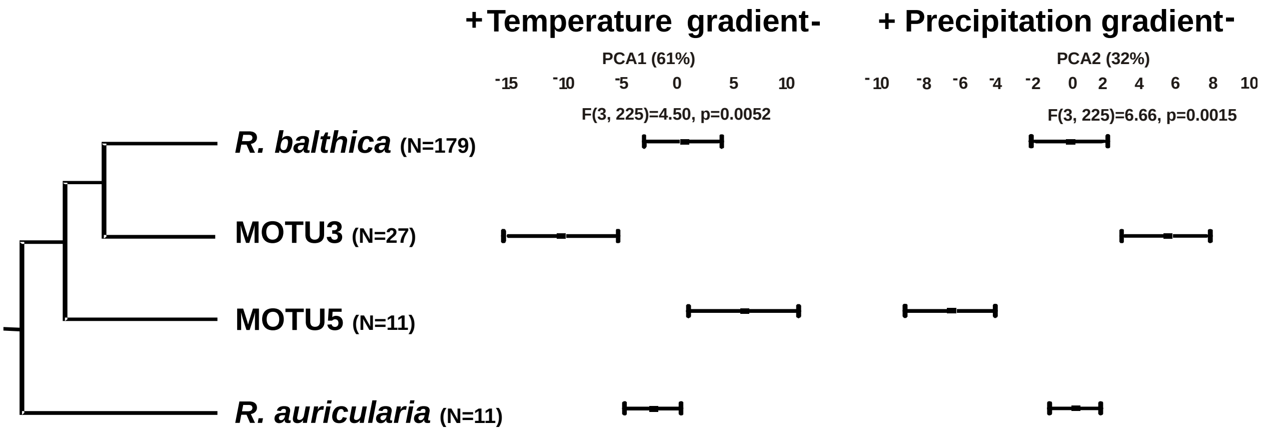

Figure S3: Realised climate niches of four *Radix* species. 95% confidence ranges of population scores along the first two principal component axes, accounting for 93% of total climate variation. The first axis is a temperature gradient, while the second axis denotes differences in overall precipitation regime. The species occupy significantly different climate niches on both axes (ANOVA, test statistics in the figure).
