# Supplementary material for: Positive selection in development and growth rate regulation genes involved in species divergence of the genus Radix
Source: BMC Evol Biol. 2015 Aug 19;15:164. doi: 10.1186/s12862-015-0434-x (PMC4539673; doi:10.1186/s12862-015-0434-x)
Supplement: Additional file 6: — Snail collection sites. (PDF 330 kb) [file 12862_2015_434_MOESM6_ESM.pdf]

Additional file 6

Snail collection sites

Species	Year	Country	Location	Latitude	Longitude
R. balthica	2007	Germany	Klingenberg, Bavaria	50.007	9.156
MOTU3	2011	France	Camplong d'aude, Languedoc-Roussillon	43.12278	2.66333
R. auricularia	2005	Germany	Oberuff, Hesse	51.035754	9.149355
MOTU5	2010	Switzerland	Lac de Retaud, de Vaud	46.359914	7.198186
